# Intracytoplasmic stable expression of IgG1 antibody targeting NS3 helicase inhibits replication of highly efficient hepatitis C Virus 2a clone

**DOI:** 10.1186/1743-422X-7-118

**Published:** 2010-06-07

**Authors:** Partha K Chandra, Sidhartha Hazari, Bret Poat, Feyza Gunduz, Ramesh Prabhu, Gerald Liu, Roberto Burioni, Massimo Clementi, Robert F Garry, Srikanta Dash

**Affiliations:** 1 Department of Pathology and Laboratory Medicine, Tulane University Health Sciences Center, 1430 Tulane Avenue, New Orleans, LA-70112, USA; 2 Department of Medicine, Gastroenterology, Tulane University Health Sciences Center, 1430 Tulane Avenue, New Orleans, LA-70112, USA; 3 Facoltà di Medicina e Chirurgia, Università Vita-Salute San Raffaele, Via Olgettina, 60 - DiBit2, 20132 Milano, Italy; 4 Department of Microbiology and Immunology, Tulane University Health Sciences Center, 1430 Tulane Avenue, New Orleans, LA-70112, USA

## Abstract

**Background:**

Hepatitis C virus (HCV) infection is a major public health problem with more than 170 million cases of chronic infections worldwide. There is no protective vaccine currently available for HCV, therefore the development of novel strategy to prevent chronic infection is important. We reported earlier that a recombinant human antibody clone blocks viral NS3 helicase activity and inhibits replication of HCV 1b virus. This study was performed further to explore the mechanism of action of this recombinant antibody and to determine whether or not this antibody inhibits replication and infectivity of a highly efficient JFH1 HCV 2a virus clone.

**Results:**

The antiviral effect of intracellular expressed antibody against the HCV 2a virus strain was examined using a full-length green fluorescence protein (GFP) labeled infectious cell culture system. For this purpose, a Huh-7.5 cell line stably expressing the NS3 helicase gene specific IgG1 antibody was prepared. Replication of full-length HCV-GFP chimera RNA and negative-strand RNA was strongly inhibited in Huh-7.5 cells stably expressing NS3 antibody but not in the cells expressing an unrelated control antibody. Huh-7.5 cells stably expressing NS3 helicase antibody effectively suppressed infectious virus production after natural infection and the level of HCV in the cell free supernatant remained undetectable after first passage. In contrast, Huh-7.5 cells stably expressing an control antibody against influenza virus had no effect on virus production and high-levels of infectious HCV were detected in culture supernatants over four rounds of infectivity assay. A recombinant adenovirus based expression system was used to demonstrate that Huh-7.5 replicon cell line expressing the intracellular antibody strongly inhibited the replication of HCV-GFP RNA.

**Conclusion:**

Recombinant human anti-HCV NS3 antibody clone inhibits replication of HCV 2a virus and infectious virus production. Intracellular expression of this recombinant antibody offers a potential antiviral strategy to inhibit intracellular HCV replication and production.

## Background

Hepatitis C virus (HCV) infection is a blood borne infectious disease that affects the liver. Only a small fraction of infected individuals clear the HCV infection naturally. In the majority of cases, the virus infection overcomes the host innate and adaptive immune responses leading to a stage of chronic infection. It has been well recognized that chronic HCV infection often leads to a progressive liver disease including cirrhosis and liver cancer. There are 170 million people representing 3% of the world's population that are chronically infected with HCV. The incidence of new infection continues to rise each year at the rate of 3-4 million [1]. Therefore, HCV infection is considered a major health-care problem worldwide. At present there is no prophylactic antibody or therapeutic vaccine available. The only treatment option for chronic HCV infection is the combination of interferon and ribavirin [2]. This therapy is not effective in clearing all chronic HCV infections. Interferon therapy is also very costly and has substantial side effects. There is a need for the development of improved antiviral therapies for the treatment of chronic HCV infection.

Hepatitis C virus is a positive-stranded RNA virus containing a single RNA genome of 9600 nucleotides in length [3]. The virus genome contains a short 341 nucleotides untranslated region (5'UTR) followed by a long open reading frame (ORF), ending with a short 3' untranslated region. The HCV genome can persist in the infected liver cells due to continuous replication of positive-stranded RNA genome. The 5' UTR of HCV RNA is crucial for the initiation of protein synthesis. This component of viral genome recognizes the host ribosome and translates HCV proteins by an IRES dependent mechanism. A single large polyprotein of 3010 amino acids is translated from the long open reading frame (ORF) encoded within the viral RNA genome. This large protein is then cleaved into 10 different individual proteins by the combined action of the cellular and viral proteases. The viral core, E1, E2, and P-7 proteins are called the structural proteins required for the production of infectious virus particles, their secretion and infection. The remaining non-structural proteins (NS2, NS3, NS4A, NS4B, NS5A, NS5B) are essential for replication of HCV positive and negative strand RNA. Among these non-structural proteins, NS3 is the viral protease and NS5B is the viral polymerase. These two proteins have been the targets of novel drug discovery [4,5]. There are now large numbers of HCV inhibitors in the clinical developments targeting these two proteins and these new drugs in combination may improve the treatment of chronic HCV infection [6].

Several novel antiviral strategies also have been developed using HCV cell culture models including antisense oligonucleotides [7-10], siRNAs [11-15], and recombinant antibodies [16-34]. Hepatitis C virus shows chronic persistent infection in the liver, even in the presence of circulating antibodies to both the structural and non-structural proteins. The vast majority of these circulating antibodies do not inhibit intracellular virus production and replication. Antibody-mediated neutralization of intracellular and extracellular virus replication and infection is a novel approach to treat chronic viral infection. The rationale of the current study is to develop an intracellular treatment approach for chronic HCV infection by using recombinant antibody technology. During the past few years, significant progress has been made in the design, selection, and production of engineered antibodies [35,36]. Antibodies can be reduced in size, rebuilt into multivalent molecules and conjugated with drugs, toxins, or radioisotopes for the treatment of cancer, autoimmune disorders, graft rejection, and infectious diseases.

We have developed a human monoclonal antibody clone derived from a chronically infected patient that effectively inhibits NS3 helicase activity [29-31]. We showed that intracellular expression of this recombinant antibody clone from a plasmid vector inhibits helicase activity and replication of HCV 1b virus [30]. Here we further explore the mechanism of action of this recombinant antibody and demonstrate that it also inhibits replication of another HCV strain. A Huh-7.5 cell expressing NS3 antibody was developed. We show that replication and infectivity of HCV 2a virus was effectively inhibited in Huh-7.5 cells expressing NS3 antibody. The replication and infectivity of HCV 2a virus was not altered in Huh-7.5 cells expressing control antibody against influenza virus.

## Materials and methods

### Cell lines and plasmid clones

Huh-7.5 cells were obtained from the laboratory of Dr. Charles M Rice (Center for the Study of Hepatitis C, The Rockefeller University, New York) and cells were cultured in Dulbecco's Modified Eagle Medium (DMEM; Invitrogen, San Diego, CA) with high glucose supplemented with non-essential amino acids, sodium pyruvate and 5% fetal bovine serum. A full-length JFH-1 clone and replicon plasmid (pSGR-JFH1) were obtained from Dr. Takaji Wakita [37] (National Institute of Infectious Diseases, Tokyo, Japan). Full-length and sub-genomic JFH1-GFP was constructed by inserting the coding sequence of green fluorescence protein in the NS5A gene described before [38].

### Development of stable Huh-7.5 cell lines expressing IgG1 antibody

The construction of plasmid vector pFab-CMV-NS3 (L+H) containing light chain, heavy chain, CH2-CH3, and part of the hinge region for a germline immunoglobulin 1 was described previously [30]. This vector allows conversion of recombinant Fab antibody into a complete IgG1 antibody in the transfected cells. Huh-7.5 cells were electroporated with 10 μg of pFab-CMV (H+L) plasmid DNA. After 24 hours, cells were selected with a growth medium containing G-418 (500 μg/ml). A control stable Huh-7.5 cell line was prepared that expressed IgG1 antibody targeted to influenza A viruses (clone Fab-9) [39]. These two Huh7.5 lines stably expressing intracellular antibody were cultured in a growth medium containing G-418 (500 μg/ml). Intracellular expression of IgG1 antibody in these two stable cell lines was confirmed by immunofluorescence microscopy. Briefly, cells cultured in chamber slides were washed with phosphate-buffered saline (PBS) pH 7.4 twice, air-dried and fixed with chilled acetone for 5 min. The cells were permeabilized by the treatment with 0.05% saponin for 10 min at room temperature. Blocking was performed with 5% fetal bovine serum (FBS) diluted in a minimum essential medium for 5 min at room temperature. The slides were washed with PBS thrice for 5 min each. The cells were incubated with goat anti-human phycoerythrin conjugated antibody (anti-human-IgG-γ chain specific-R-Phycoerythrin, Sigma-Aldrich, Saint Louis, MO) at 1:50 dilution (in DMEM+5% FBS) for 1 hour. When staining was completed, the slides were washed three times with PBS and mounted with hoechst dye (H33342, Calbiochem, Darmstadt, Germany) at a concentration of 10 μg/ml prepared in water containing 50% glycerol. Finally, the slides were examined under a fluorescence microscope at 563 nm for the red fluorescence and 340 nm for blue fluorescence. For each area, two sets of pictures were generated. Superimposing blue with red fluorescence using the Abode Photoshop computer software (V 7.0) generated the final image.

### Replication Assay

The effect of an intracellular antibody expression in Huh-7.5 cells on the replication of full-length JFH1-GFP RNA genome was examined by using a transfection based replication assay. Full-length *in vitro *HCV-GFP RNA transcripts were prepared from *XbaI *digested linearized pJFH1-GFP plasmid by using a commercially available MEGA script kit (Ambion Inc, Austin, TX). The HCV RNA pellet was re-suspended in nuclease free water and 20 μg aliquots of this RNA was stored at -80°C. Approximately, 2 × 10^7 ^cells were re-suspended in 400 μl of serum free DMEM, mixed with 20 μg of *in vitro *transcribed RNA and was electroporated using a Gene Pulser Xcell apparatus (Bio-Rad Laboratories Inc. Hercules, CA) with the condition 260 V, 960 μF. Following this step, cells were cultured in DMEM with 10% fetal bovine serum. The expression of GFP due to HCV replication was monitored under a fluorescence microscope (Olympus 1 × 70) with every 24 hours interval and the images were captured using an Olympus DP-71 digital camera. Positive- and negative-strand HCV-RNA in the transfected cells was detected by ribonuclease protection assay (RPA). Briefly, total RNA was isolated from the HCV transfected cells every 24 hours by the GITC method and subjected to RPA using a probe targeted to the 5'UTR of HCV. The same amounts of the RNA extracts were subjected to RPA for GAPDH mRNA. To detect HCV RNA, we prepared a plasmid construct called pCR-II-NT-218, which have the sequence of 79-297 nucleotides of 5'UTR sequence of the JFH-1 clone. This plasmid was linearized with *HindIII *enzyme and positive strand RNA probe was prepared using T7 RNA polymerase to detect the HCV negative strand RNA. Likewise, this plasmid was linearized with the *Xba I *restriction enzyme and Sp6 RNA polymerase was used to prepare a negative-strand RNA probe for the detection of a positive-strand HCV RNA. We used a linearized plasmid pTRI-GAPDH-human antisense control template was used to prepare probe to detect GAPDH mRNA using Sp6 RNA polymerase (Ambion Inc., Austin, TX).

### Infectivity Assay

The effect of intracellular antibody expression on production of infectious HCV was examined by multicycle infectivity assay [38]. Huh-7.5 cells were transfected with 20 μg of in vitro transcribed full-length JFH1-GFP RNA by electroporation method. After 96 hours, cells were collected by scraping and lysed by four rounds of freeze-thaw cycles. The cell lysates were clarified by centrifugation at 3400 rpm for five minutes. The clear supernatant was collected and titer of HCV in the supernatant was determined by real-time RT-PCR using a primer set targeted to the 5'UTR. Tissue culture infective dose (TCID50 and MOI) of the virus stock was determined using 10-fold serial dilution of the virus containing supernatant using 2-well Lab-Tek chamber slides (Nalge-Nunc International, Rochester, New York). Huh-7.5 cells stably expressing intracellular antibody were seeded at a density of 1 × 10^6 ^cells/100 mm plate. The next day the culture medium was removed and cells were infected with 3 ml of culture supernatant containing infectious virus (5 × 10^5 ^virus particles/ml, MOI 1.5). After overnight incubation, the cells washed three times using 10 ml of PBS and incubated with 10 ml of complete growth medium. Cell free culture supernatants were collected after 96 hours, clarified by centrifugation. Three ml of culture supernatants was used to infect new batch of antibody expressing Huh-7.5 cells. The infectivity assay was performed up to four cycles each using the identical procedure. At the end of infectivity assay, RNA was isolated from 1 ml of culture supernatants and HCV RNA titer was measured by real-time RT-PCR.

### Real time reverse transcription polymerase chain reaction (RT-PCR)

Real time RT-PCR was performed to quantify HCV RNA levels in the infected cell culture using a published protocol [40]. The 243 bp HCV DNA was amplified from the RNA extract by reverse transcription polymerase chain reaction using the outer sense (OS) primer 5'-GCAGAAAGCGCCTAGCCATGGCGT-3' (67-90) and outer anti-sense (OAS) primer 5'-CTCGCAAGCGCCCTATCAGGCAGT-3' (287-310). First the complementary DNA synthesis was performed from positive-strand HCV-RNA using an outer anti-sense primer (OAS) targeted to the highly conserved 5'UTR region of HCV in 20 μl volume. Briefly, 2 μg of total cellular RNA were mixed with 1 μl OAS primer (200 ng/μl), denaturized at 65°C for 10 minutes and annealed at room temperature. Avian myeloblastosis virus (AMV) reverse transcriptase (10 U) (Promega, Madison, WI) was added and incubated at 42°C for 60 minutes in the presence of 50 mmol/L Tris, pH 8.3, 50 mmol/L ethylenediaminetetraacetic acid (EDTA), 500 nmol/L dNTP, 250 nmol/L spermidine, and 40 U RNasin (Promega, Madison, WI). The cDNA was stored at -20°C until use. SYBR Green real time PCR amplification was performed in 20 μl of volume containing 10 μl of SYBR Green ER qPCR SuperMix, 1 μl (250 ng/ul) of sense and antisense primer with 4 μl of cDNA and 4 μl of distilled water. All samples were run in triplicate. The amplification was carried out using the standard program recommended by Bio-Rad Laboratory that includes: 50°C for 2 minutes, 95°C for 8 minutes, then additional 50 cycles wherein each cycle consists of a denaturation step at 95°C for 10 seconds, and annealing and extension step at 60°C for 30 seconds. At the end of the amplification cycles, melting temperature analysis was performed by a slow increase in temperature (0.1°C/s) up to 95°C. Amplification, data acquisition, and analysis were performed on CFX96 Real-Time instrument using CFX manager software (Bio-Rad, Hercules, CA).

### Construction of adenovirus vector containing IgG1 antibody

A replication defective adenovirus construct carrying the gene for the recombinant human antibody (Ad-IgG1) was prepared using standard PCR and cloning methods. We used the pFab-CMV-NS3 (L+H) plasmid construct to prepare a recombinant adenovirus vector (30). The cloning process was carried out in multiple steps. First, the heavy chain antibody expression cassette containing the CMV promoter-antibody ORF, Fd termination sequence and heavy chain polyadenylation sequences were PCR amplified and assembled in pGEM-7Z(f+) vector (Promega, Madison, WI) using *XhoI *and *BamHI *sites. An unique *HindIII *site was introduced before the *XhoI *and *BamHI *site such that the entire antibody expression cassette can be excised from the pGEM-7Z(f+) plasmid and inserted into the adenovirus pShuttle Vector (QBIOgene, AES1020, without CMV promoter) using a unique *HindIII *site. After cloning, the exact orientation of the heavy chain antibody expression cassette was confirmed by DNA sequence analysis. At the second step, the antibody light chain gene was inserted into the same plasmid shuttle (pShuttle vector without CMV promoter) using two PCR fragments. The first light chain CMV promoter-leader sequence and light chain ORF was cloned into a pShuttle plasmid using *Kpn1 *and *XbaI *sites. Then the remaining light chain termination sequence and polyadenylation sequences were introduced into the same pShuttle vector using *XbaI *sites. The orientation of the CMV promoter of the light chain and heavy chain reading frame in the resulting plasmid was confirmed by restriction analysis. The recombinant plasmid is called pShuttle CMV NS3 (H+L). The antiviral effect of recombinant antibody clone was confirmed again by transfection into a replicon cell line. The pShuttle CMV NS3 (H+L) antibody gene was then transferred into the adenoviral genome (pAdEasy, QBiogene, Carlsbad, CA) by homologous recombination. The recombinant adenovirus plasmid was examined by restriction digestion analysis. The recombinant adenovirus plasmid was linearized with a *PacI *restriction enzyme and transfected to QBI-293 cells (QBiogene, Carlsbad, CA). After several weeks, the recombinant adenovirus was plaque purified and amplified on 293 cells. Large-scale purification of the recombinant adenovirus was performed by CsCL gradient centrifugation. The titer of recombinant adenovirus (virus particles/ml) was determined by using absorbance at optical density at 260 nm, a standard protocol supplied in the kit. A recombinant adenovirus carrying firefly luciferase (Ad-Luc) was used as a control to exclude the non-specific effect of recombinant adenovirus infection on HCV replication.

### Immunocytochemistry

Huh-7.5 cells were cultured in chamber slides and after 24 hours they were infected with a different concentration of recombinant adenovirus (Ad-IgG1). The intracellular expression of a recombinant antibody was examined by an immunostaining method using goat anti-human antibody (Sigma-Aldrich, Saint Louis, MO). Briefly, Huh-7.5 cells cultured in chamber slides were washed with phosphate-buffered saline (PBS) pH 7.4 twice, air-dried and fixed with chilled acetone for five minutes. The cells were permeabilized by the treatment with 0.05% saponin for 10 minutes at room temperature. Blocking was performed with 5% normal goat serum diluted in a minimum essential medium for 30 minutes at room temperature. Blocking for endogenous biotin-avidin was performed using an avidin/biotin blocking kit (Vector Laboratories Inc., Burlingame, CA). Blocking for endogenous peroxidase was carried out with 0.9% H_2_O_2 _for 30 minutes at room temperature. The cells were incubated with a biotin conjugated goat anti-human IgG antibody (1:500 dilution, Sigma-Aldrich, Saint Louis, MO). The slide was then washed three times and incubated with an anti-mouse biotin conjugated antibody (1:1000) for one hour at room temperature. The slides were then washed and incubated for 30 minutes with Elite avidin-biotin peroxidase complex (Vector Laboratories Inc., Burlingame, CA). The slides were reacted with diaminobenzidine for 10 minutes. Counterstaining was performed with hematoxylin for one minute. After dehydration, the slides were mounted with per mount and observed by light microscopy.

### Western blotting

To make sure that the intracellular expressed antibody molecule processed accurately, western blot analysis was performed using lysates prepared from the adenovirus infected Huh-7.5 cells. Immunoblotting was performed with a peroxidase conjugated rabbit anti-human antibody at a dilution of 1:500. (Sigma-Aldrich, Saint Louis, MO). The membrane was developed using the enhanced-chemiluminescence detection kit (ECL kit) (Amersham Pharmacia, Piscataway, NJ) and was exposed to chemiluminescent-sensitive film (Kodak Rochestor, NY).

## Results

### Huh-7.5 cells stably expressing NS3 antibody inhibits HCV 2a replication

**Figure 1:**
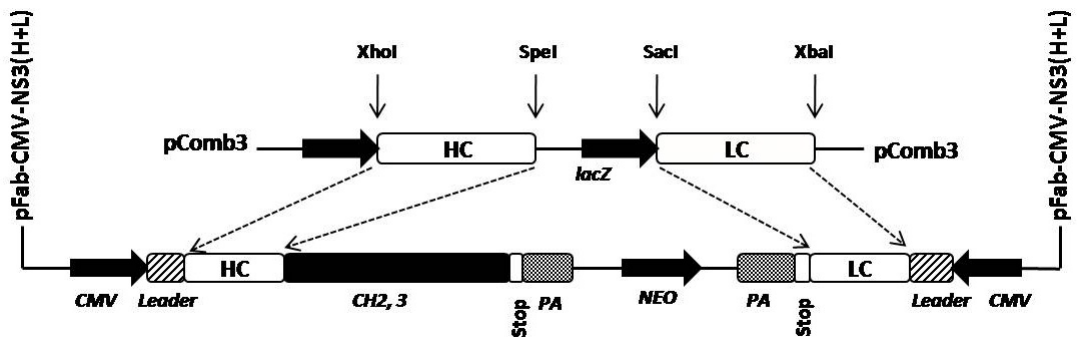
**The schematic diagram of the recombinant antibody construct**. The recombinant Fab antibody clone was expressed in mammalian cells using the expression vector (pFab-CMV-NS3 (H+L) that has two identical CMV promoters. This vector was used to generate stable Huh-7.5 cells expressing intracellular IgG1 antibody.

**Figure 2:**
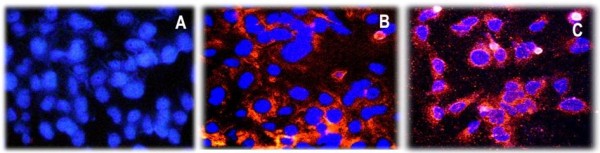
**Immunofluorescence staining showing stable intracytoplasmic expression of NS3 helicase specific antibody and control antibody in Huh-7.5 cells**. Stable Huh-7.5 cells were seeded in 2-well chamber slides and after 48 hours cells were stained with phycoerythrin conjugated anti-human IgG1 antibody at a dilution of 1:50. After this step, cells were counterstained with hoechst dye. **A**: Negative staining of control Huh 7.5 cells. **B: **Huh 7.5 cells stably expressing a control antibody. **C: **Huh 7.5 cells stably expressing NS3 antibody.

**Figure 3:**
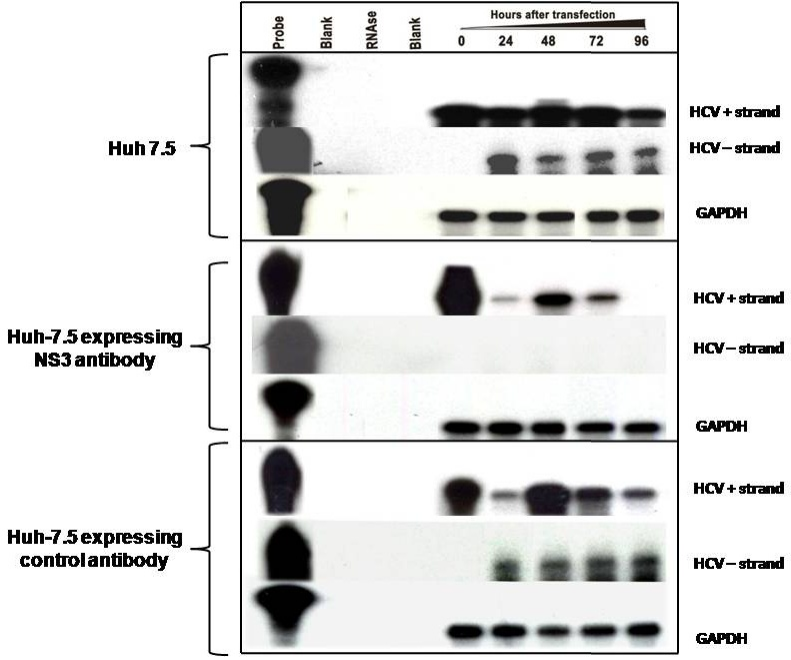
**HCV positive- and negative-strand RNA detection by RPA showing the complete inhibition of HCV replication in stable Huh 7.5 cells expressing NS3 antibody (middle panel)**. Huh-7.5 cells were transfected with *in vitro *transcribed full-length HCV-GFP RNA. Cells were harvested after 0, 24, 48, 72 and 96 hours of post transfection. Total RNA was isolated and subjected to positive- and negative-strand HCV RNA detection and GAPDH mRNA by RPA. **Upper panel: **shows HCV positive- and negative-strand HCV RNA levels in Huh-7.5 cells transfected with full-length HCV-GFP mRNA. **Middle panel: **inhibition of HCV positive- and negative-strand RNA levels in Huh-7.5 cells expressing NS3 antibody. **Lower panel: **shows HCV positive- and negative-strand HCV RNA levels in Huh-7.5 cells expressing control antibody.

**Figure 4:**
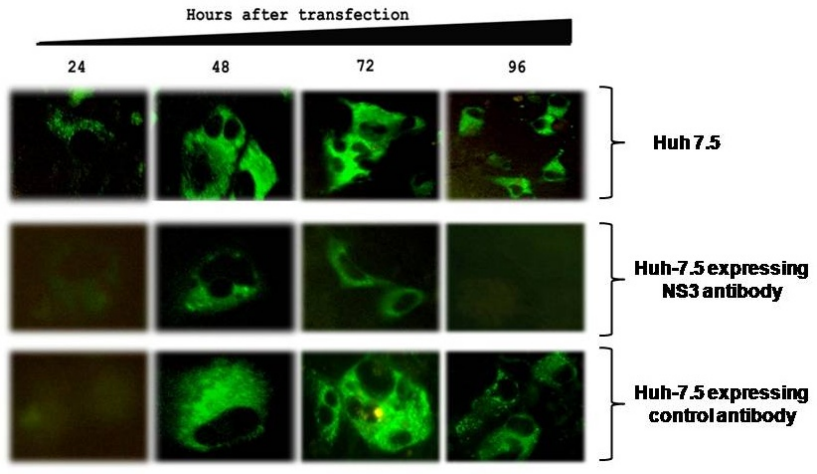
**Expression of GFP fusion protein in the Huh-7.5 cells expressing intracellular NS3 antibody and control antibody**. Huh-7.5 cells were transfected with *in vitro *transcribed full-length HCV-GFP RNA. Cells were examined for GFP expression after 0, 24, 48, 72 and 96 hours of post transfection. **Upper panel: **shows HCV-GFP expression in Huh-7.5 cells transfected with full-length HCV-GFP mRNA. **Middle panel: **shows HCV-GFP expression was terminated after 96 hours in Huh-7.5 cells expressing NS3 antibody. **Lower panel: **shows no reduction in the level of HCV-GFP expression in Huh-7.5 cells expressing control antibody.

**Figure 5:**
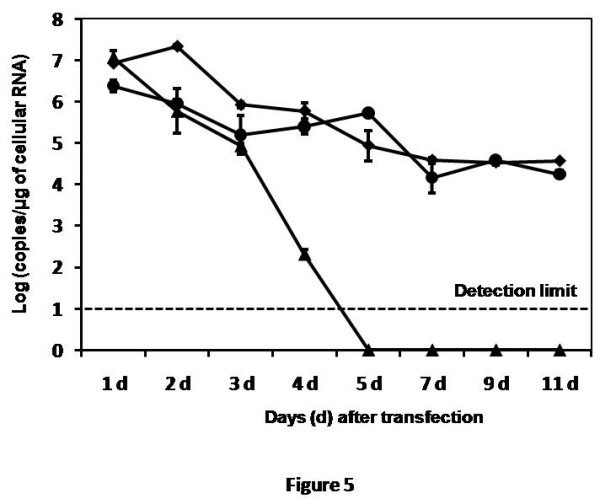
**HCV RNA levels in Huh-7.5 cell lines after full-length HCV-GFP RNA transfection determined by real-time RT-PCR**. Stable Huh-7.5 cells were transfected with *in vitro *transcribed full-length HCV-GFP RNA. Cells were harvested after 0, 24, 48, 72 and 96 hours of post transfection. Total RNA was isolated and subjected real-time PCR. (black diamond) Huh-7.5 only, (black triangle) Huh-7.5 cells expressing NS3 antibody, (black circle) Huh-7.5 cells expressing control antibody.

The initial step of this study was the development of a Huh-7.5 cell line with stable expression of intracellular antibody. The recombinant human antibody gene was introduced in to a mammalian plasmid expression vector that expresses the heavy and light chain antibody gene using the two identical CMV promoters (Fig. 1). This allows production of equal amounts of heavy and light chains of the antibody molecule in the cytoplasm. A stable Huh-7.5 cell line expressing NS3 antibody targeting the HCV NS3 helicase was prepared. Another Huh-7.5 cell line stably expressing antibody against influenza virus was used as a control. We show that both the Huh-7.5 cell lines expresses IgG1 antibody in the cytoplasm by immunological staining (Fig. 2). Stable intracellular antibody expression ensured no cellular toxicity since these cells can be propagated in culture for prolonged time. To study the effect of the intracellular antibody on full-length HCV RNA replication, we used a chimeric full-length JFH1-GFP clone where the coding sequence of GFP was introduced in the NS5A region [38]. We have shown that this clone replicates in Huh-7.5 cells and produces positive- and negative-strand HCV RNA by ribonuclease protection assay. To determine the antiviral effect of intracellular antibody expression on the replication of full-length JFH-1 clone, full-length JFH1-GFP HCV-RNA was transfected to stable Huh-7.5 cell lines expressing an intracellular antibody. The effect of intracellular antibody expression on replication of HCV was determined by RPA assay at 0, 24, 48, 72 and 96 hours. The results of RPA assay for the detection of positive- and negative-strand RNA detection are shown in Fig. 3. The levels of positive-strand RNA decreased over time and remained undetectable at 96 hours only in the cells expressing NS3 antibody. In contrast, positive-strand HCV RNA was detected at all time points in cells expressing an unrelated control antibody or Huh-7.5 cells without antibody expression. HCV is a positive-strand RNA virus that replicates by a negative-strand RNA intermediate. The effect of antibody expression on the replication of JFH1-GFP RNA in Huh-7.5 cells was examined by measuring HCV negative-strand RNA levels by RPA. HCV negative-strand RNA remained undetected in Huh-7.5 cells expressing NS3 antibody. In contrast, HCV negative-strand RNA was detected in Huh-7.5 cells expressing control antibody and Huh-7.5 cells without antibody expression. These results suggest that HCV replication was terminated in cells stably expressing intracellular helicase antibody, while in control cells HCV replication was unaltered. The antiviral effect of intracellular helicase antibody was examined by green fluorescence protein expression in a kinetic study. The expression of HCV-GFP chimera protein specifically decreased in cells expressing NS3 antibody and the protein expression remained undetectable at 96 hours (Fig 4). The level of viral RNA in the antibody expressing cells and control Huh-7.5 cells after transfection was quantitated by using real-time RT-PCR using a primer set targeted to the highly conserved 5'UTR region. HCV RNA levels progressively decreased in Huh-7.5 cells expressing NS3 antibody (Fig. 5). There are significant differences in the levels of HCV RNA between the NS3 antibody expressing cells and two control cell lines. Taken together these results indicate that full-length JFH1-GFP RNA replication was selectively inhibited in Huh-7.5 cells expressing intracellular NS3 antibody.

### Huh-7.5 cells stably expressing NS3 antibody inhibits production of intracellular and extracellular virus

**Figure 6:**
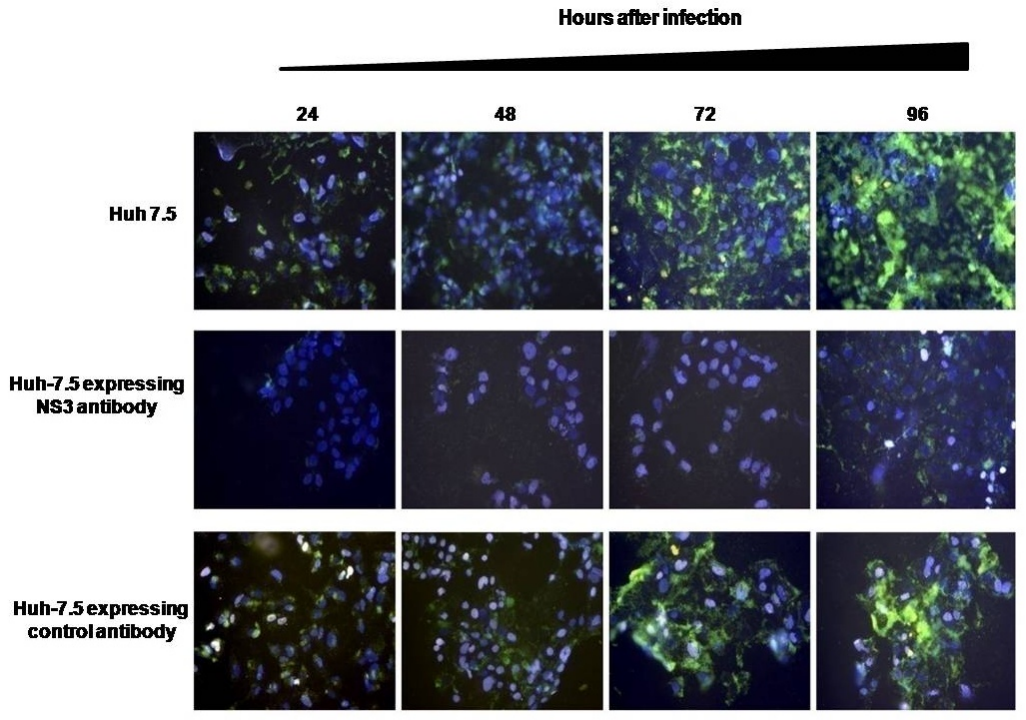
**Infectivity assay of HCV-GFP chimera virus in Huh-7.5 cells expressing NS3 antibody and control antibody**. Huh-7.5 cells (1 × 10^4^) were seeded on a 2-well glass chamber slide, next day infected with JFH1-GFP virus for overnight at 37°C. Infected cells were examined for GFP expression after 24, 48, 72 and 96 hours of post infection. Superimposing blue (nucleus) with green fluorescence (HCV GFP) using the Abode Photoshop computer software generated the final image. **Upper panel: **shows HCV-GFP expression in Huh-7.5 cells infected with JFH1 virus. **Middle panel: **shows HCV-GFP expression was terminated in Huh-7.5 cells expressing NS3 antibody. **Lower panel: **shows no reduction in the level of HCV-GFP expression in Huh-7.5 cells expressing control antibody.

**Figure 7:**
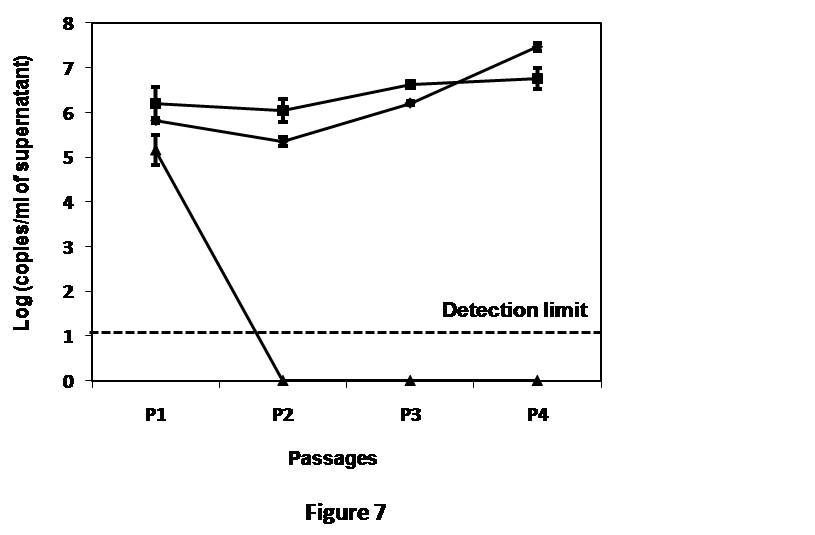
**The production of infectious HCV particles in the Huh-7.5 cells expressing NS3 antibody and control antibody**. 1 × 10^6 ^cells were seeded in a 100-mm plate was infected with HCV-GFP virus at a MOI of 1.5. Cell free supernatants were collected every 96 hours. Three milliliter of culture supernatants was used to infect freshly cultured Huh-7.5 cells. The infectivity assay was repeated for four passages. The production of infectious HCV using RNA extracts from 1 ml of culture supernatants was determined by real-time RT-PCR. (black diamond) Huh-7.5 only, (black triangle) Huh-7.5 cells expressing NS3 antibody, (black square) Huh-7.5 cells expressing control antibody.

The effect of intracellular NS3 antibody on the production of infectious virus particles was examined by performing multicycle infectivity assay. We previously established infectivity assay for HCV using the JFH1-GFP clone. The infectivity of HCV can be directly determined by examining the expression of GFP under a fluorescence microscope. Huh-7.5 cells expressing NS3 antibody and Huh-7.5 cells expressing control antibody were infected with infectious HCV and replication of HCV-GFP chimera after natural infection was examined in a kinetic study. We found that the replication of HCV-GFP chimera virus was inhibited in the Huh-7.5 cells expressing NS3 antibody since we did not see any GFP expression (Fig. 6). In contrast, HCV-GFP expression in the infected cells was detected in both the control Huh-7.5 cells. The infectivity assay was serially repeated an additional three cycles using 3 ml of culture supernatants (MOI 1.5). The production of infectious HCV in Huh-7.5 cells with or without antibody expression was measured by real-time RT-PCR. The results of these experiments (Fig. 7) indicate that there is a significant drop in the HCV RNA levels after first passage in Huh-7.5 cells expressing NS3 antibody. The levels of HCV RNA in the supernatants did not change in Huh-7.5 cells and Huh-7.5 cells expressing control antibody. These results clearly indicate that the NS3 antibody prevents intracellular HCV RNA replication leading to reduced virus particle formation and secretion.

### Co-localization of intracellular antibody expression with HCV in replicon cells

**Figure 8:**
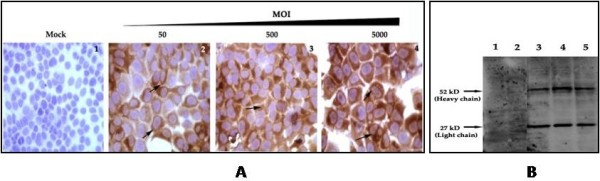
**Intracytoplasmic expression of IgG1 antibody delivered by adenovirus vector**. Left panel shows dose-dependent intracytoplasmic expression of IgG1 antibody in Huh7.5 cells delivered by adenovirus vector. Huh-7.5 cells were infected with increasing concentrations of Ad-IgG. After 24 hours, intracellular antibody expression was examined by immunostaining with a biotin conjugated goat anti-human IgG1 antibody. Slides were then counterstained with hematoxylin. **1**: Mock infected cells showing negative staining. **2-4: **Dose dependent intracytoplasmic brown staining was seen in majority of Huh-7.5 cells infected with Ad-IgG virus **(A)**. Western blot analysis showing the presence of light chain and heavy chain antibody in the lysates of Huh-7.5 cells infected with Ad-IgG **(B)**. Huh-7.5 cells were infected with three different batches of recombinant Ad-IgG at a concentration of 4000 MOI. Cell lysates were made after 24 hours. 50 μg of protein lysates were separated on a 10% SDS PAGE and transferred to a nylon membrane. Immunoblotting was performed with peroxidase conjugated rabbit anti-human antibody. The membrane was developed using the ECL kit and was exposed to chemiluminescent-sensitive film. **1**. Uninfected Huh-7.5 cells. **2**. Huh-7.5 cells infected with an adenovirus luciferase (Ad-Luc). **3-5**. Huh-7.5 cells infected with three different batches of plaque-purified virus (Ad-IgG). Two specific bands at molecular weight 27 kD and 52 kD represent the light chain and the heavy chain of the IgG1 antibody respectively.

**Figure 9:**
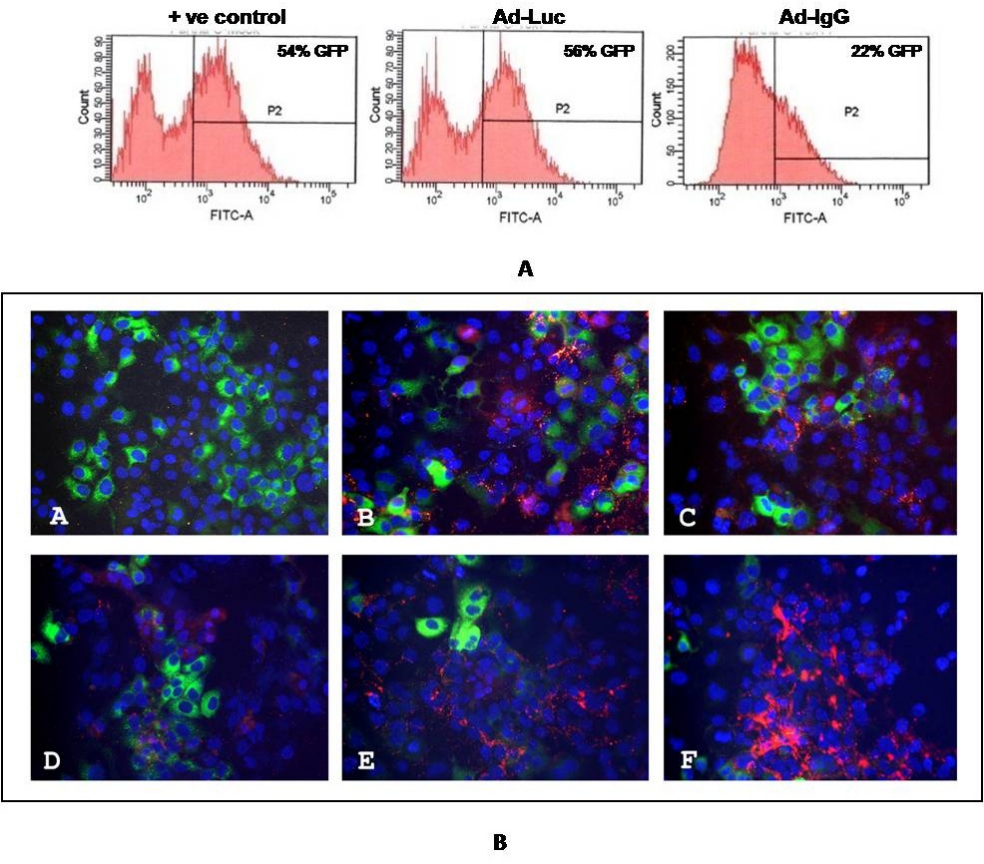
**Intracellular antibody expression by adenovirus vector inhibits HCV replication in a sub-genomic GFP based stable replicon cell line**. **Upper Panel **shows flow cytometric analysis of HCV-GFP protein level in replicon cell line expressing intracellular NS3 antibody from adenovirus vector. 1 × 10^6 ^replicon cells were seeded in a 100 mm tissue culture dishes and next day they were infected with recombinant adenovirus (Ad-IgG or Ad-Luc). After 72 hours, cells were harvested by using trypsin-EDTA and then analyzed by a flow cytometer where GFP expressing cells were quantified. **Lower panel **shows that intracellular antibody expression eliminates HCV GFP expression in replicon cell line in a co localization experiment. Replicon cells (2 × 10^4^) were grown in a 2-well chamber slide and after 24 hours cells were infected with increasing concentration of Ad-IgG (ranged from 50, 500, 1000, 2000 and 4000 MOI). After 72 hours, cells were fixed for 15 min with 2% paraformaldehyde. This fixation did not destroy GFP expression. Intracellular expression of antibody was performed using phycoerythrin conjugated anti-human IgG1 antibody (1:50 dilution). After this step, cells were washed and counterstained with hoechst dye. Multiple areas in the slide were randomly examined. Three sets of pictures from the same area were taken. First, the intracellular antibody expression (red), second for the GFP expression and third blue nuclear staining of stained cells under a fluorescent microscope. Finally, the combined picture in the same area was composed using Abode Photoshop (version 7.0) computer software. **A: **Shows fairly high-level expression of HCV GFP the expression in S-3/GFP replicon cells. **B-F: **Co-localization of GFP and intracellular antibody. The expression of GFP was decreased in cells infected with Ad-IgG and panel (B-E) the co-localization HCV-GFP (green) and intracellular antibody (red).

To demonstrate intracellular antibody expression delivered by adenovirus, Huh-7.5 cells were infected with different doses of Ad-IgG1 and intracellular antibody expression was determined by immunocytochemistry. A very distinct bright immunostaining pattern was observed suggesting high levels expression of antibody in the entire cytoplasm but not in the nucleus (Fig. 8A). None of the control cells showed staining suggesting that high level and dose dependent expression of antibody can be achieved using Ad-IgG1. Adenovirus mediated delivery of the antibody appears to be efficient since most of the cells in the culture showed a dose dependent expression of the intracellular antibody. To determine if intracellular antibody molecules expressed are processed accurately, Western blot analysis was performed using the Ad-IgG1 infected Huh-7.5 cell lysates with goat-antibodies that specifically bind to the Fab portion of a human IgG1 molecule (Fig. 8B). Two bands that correspond to the molecular weight of heavy and light chain of antibody molecule were detected in the extracts of Ad-IgG1 infected Huh-7.5 cells but not in the mock infected cells. These results suggested that antibody molecules are accurately processed and expressed high levels in the cytoplasm of a liver derived cell line. In order to determine the extent of inhibition of HCV replication that occurred due to intracellular expression of the antibody in the cytoplasm of individual replicon cells, we performed co-localization experiments using a GFP-tagged replicon cell line. This cell line supports high levels of replication of HCV JFH1-sub genomic RNA that can be directly visualized by GFP expression. This S-3/GFP replicon cell was infected with Ad-IgG1 virus. After 72 hours, the antiviral efficacy of intracellular antibody expressed from recombinant adenovirus vector was examined by flow cytometric analysis (Fig. 9A). The S-3/GFP replicon cells expressed high level of GFP that can be detected by flow analysis. The expression of GFP was not altered when infected with Adv-Luciferase. The GFP expression was inhibited in S-3/GFP replicon cells infected with Adenovirus carrying NS3 antibody gene. We then performed a co-localization experiment using the S-3/GFP cells where intracellular antibody expression was examined using a phycoerythrin-labeled goat-anti-human IgG1 antibody and HCV was detected by GFP expression. Slides were randomly examined for antibody expression as well as GFP expression under a fluorescence microscope and fluorescence pictures in the same areas were composed. We anticipate that if the antibody expression inhibits HCV replication then one would expect not to see GFP expression in the same replicon cells that express intracellular antibody (Fig. 9B-F), suggesting that cells expressing NS3 antibody showed negative expression for GFP. Likewise cells showing GFP expression did not show antibody expression. We did not observe hepatocytes showing positive expression of both HCV (GFP) as well as intracellular antibody (red) in the entire culture. These results suggest that intracellular antibody expression in individual replicon cells resulted in the inhibition of GFP expression.

## Discussion

We described here a novel intracellular treatment approach for chronic HCV infection using a humanized recombinant antibody targeting the viral NS3 helicase enzyme. We hypothesized that intracellular expression of the antibody targeting the viral NS3 helicase should inhibit its enzyme activity and HCV replication. The NS3 helicase enzyme is crucial for the HCV genome replication justifying it as potential target for ant-viral therapy [41-43]. As a proof-of-principle, we have already shown that transient transfection of this human antibody clone using a plasmid vector into HCV 1b replicon cell lines inhibit HCV helicase activity and HCV RNA replication [30]. The replicon-based model lacks the structural proteins and does not produce infectious virus. Therefore we performed this study to determine the success of inhibiting full-length virus genome replication using this antibody clone.

The antiviral property of this antibody clone was examined using the highly efficient JFH1 clone belonging to HCV 2a virus strain. This clone has now been widely used by a number of investigatons to study the replication of full-length virus genome after natural infection using Huh-7.5 cell culture. For this reason, stable Huh-7.5 cell lines were prepared that express HCV NS3 specific antibody and influenza specific control antibody. Huh-7.5 cells expressing intracellular antibody were cultured using a medium containing G-418 selection. We showed that Huh-7.5 cell expresses a recombinant antibody by immunocytochemical staining. To study the effect of antibody expression on HCV RNA replication, the HCV-GFP chimeric clone was transfected to Huh-7.5 cells stably expressing either NS3 helicase antibody or a control recombinant antibody. It was shown that HCV positive-strand RNA, negative-strand RNA levels and NS5A-GFP fusion protein expression was inhibited in Huh-7.5 cells expressing intracellular antibody. The inhibition of viral HCV RNA replication and protein expression was not seen in control Huh-7.5 cells. The level of viral RNA replication was not altered in two other control cell lines, including those expressing an antibody to influenza virus, suggesting that this effect is specific to NS3 helicase antibody expression.

The inhibition of infectious virus particle production in stable antibody expressing Huh-7.5 cell line was examined using an infectious cell culture system for over four cycles of infectivity assay. We showed that the production of HCV in the NS3 antibody expressed Huh-7.5 cells was strongly inhibited and the titer of HCV remained below the detection limit after first passage. The infectious HCV particle production in two control Huh-7.5 cells were maintained at a relatively high levels in the range of 10^7^-10^5 ^copies of HCV RNA/ml in the culture supernatants. The results of multicycle infectivity assay support the notion that intracellular NS3 antibody effectively suppressed the virus replication and infectious virus production. The intracellular antibody expression appears to have effectively suppressed the production of infectious HCV. In this study no evidence of escape mutant HCV was observed over four passages. We showed that most of the cells expressed antibody intracellular and inhibited HCV expression by co-localization studies. We showed that the antibody gene is accurately processed and expressed at high levels using an adenovirus vector.

Recombinant antibody-based antiviral strategies for HCV have been reported by a number of investigators. Some of the recombinant antibodies are directed against structural proteins [17,18,22,23,26-28,32]. Antibodies targeted against E1 and E2 regions can be utilized to neutralize HCV infection to cells *in vitro *as well as *in vivo*. Likewise, it is expected that intracellular expression of antibodies targeting the viral core protein should interfere with formation of infectious virus production and packaging. Some investigators have also developed recombinant antibodies targeting the viral non-structural proteins (NS3 protease, helicase and polymerase) of HCV [20,24,25,29-31]. These antibodies should be used to effectively block the viral protease, helicase and polymerase activity and thereby inhibit intracellular HCV replication and infection. One of the major problems in this antiviral strategy is the intracellular delivery of the antibody for therapeutic approaches in human. As a proof of principle we have demonstrated that intracellular inhibition of replication of the highly efficient JFH-1 virus clone can be achieved using an antibody clone targeted to the NS3 helicase. We also showed that the intracellular expression of a recombinant antibody to a liver derived cell line efficiently inhibits JFH-1 virus replication and production. The replication-defective adenovirus expresses high levels of therapeutic antibody in cultured cells. This will allow large-scale antibody purification from mammalian cells in culture. We realize that the use of adenovirus has considerable adverse side effects in humans; therefore, future research will focus on developing safer non-viral delivery methods of targeted delivery antibody to liver cells to inhibit HCV replication.

## Competing interests

The authors declare that they have no competing interests.

## Authors' contributions

PKC performed most of the biochemical experiments, participated in the design of the study and wrote the initial draft of the manuscript. SH, RP and GL contributed to construct the replication defective adenovirus carrying the recombinant antibody gene. BP and FG helped in some biochemical experiments. RB, MC supplied the recombinant antibody clones and helped to prepare the manuscript. RFG and SD supervised, helped to design the study and finally edited the manuscript. All authors read and approved the final manuscript.
